# The Effects of Laser-Assisted Winding Process Parameters on the Tensile Properties of Carbon Fiber/Polyphenylene Sulfide Composites

**DOI:** 10.3390/ma17184664

**Published:** 2024-09-23

**Authors:** Hongbo Geng, Xuewen Cao, Lei Zu, Helin Pan, Guiming Zhang, Qian Zhang, Jianhui Fu, Lichuan Zhou, Qiaoguo Wu, Xiaolong Jia, Honghao Liu

**Affiliations:** 1 School of Mechanical Engineering, Hefei University of Technology, Hefei 230009, China; 2022020005@mail.hfut.edu.cn (H.G.); zulei@hfut.edu.cn (L.Z.);; 2 Anhui Province Key Lab of Aerospace Structural Parts Forming Technology and Equipment, Hefei University of Technology, Hefei 230009, China; 3 Engineering Research Center of Advanced Composite Materials Design & Application of Anhui Province, Hefei 230051, China; 4 School of Civil and Hydraulic Engineering, Hefei University of Technology, Hefei 230009, China; 5 State Key Laboratory of Organic-Inorganic Composites, College of Materials Science and Engineering, Beijing University of Chemical Technology, Beijing 100029, China

**Keywords:** CF/PPS, laser heating, winding molding, process parameters, optimization analysis

## Abstract

Currently, there is limited research on the in situ forming process of thermoplastic prepreg tape winding, and the unclear impact of process parameters on mechanical properties during manufacturing is becoming increasingly prominent. The study aimed to investigate the influence of process parameters on the mechanical properties of thermoplastic composite materials (CFRP) using laser-assisted CF/PPS winding forming technology. The melting point and decomposition temperature of CF/PPS materials were determined using DSC and TGA instruments, and based on the operating parameters of the laser-assisted winding equipment, the process parameter range for this fabrication technology was designed. A numerical model for the temperature of laser-heated CF/PPS prepreg was established, and based on the filament winding process setup, the heating temperature and tensile strength were simulated and tested. The effects of process parameters on the heating temperature of the prepreg and the tensile strength of NOL rings were then analyzed. The non-dominated sorting genetic algorithm (NSGA-II) was employed to globally optimize the process parameters, aiming to maximize winding rate and tensile strength. The results indicated that core mold temperature, winding rate, laser power, and their interactions significantly affected mechanical properties. The optimal settings were 90 °C, 418.6 mm/s, and 525 W, achieving a maximum tensile strength of 2571.51 MPa. This study provides valuable insights into enhancing the forming efficiency of CF/PPS-reinforced high-performance engineering thermoplastic composites.

## 1. Introduction

Due to their excellent mechanical properties, carbon fiber-reinforced polymer composites (CFRP) with thermoplastic resins have become a hot research topic. Compared to common composites, thermoplastic composites offer significant advantages such as being lightweight, and having high strength, fatigue resistance, and design flexibility. Additionally, they have a longer storage life at room temperature and can be repeatedly recycled and reprocessed [1]. However, the high viscosity and melting points of thermoplastic resins pose considerable challenges during the in situ molding process, requiring careful optimization to achieve high-performance results.

In order to obtain high-performance thermoplastic composites, researchers have conducted studies on the effects of process parameters on the mechanical properties of structures for some common molding processes, such as lay-up molding [2,3,4,5,6]. Oromiehie E. et al. [7] examined how automated fiber placement (AFP) process parameters including compaction force, heat source temperature, and filament laying speed affect laminate interlaminar shear strength (ILSS) using a hot gas torch. They found that these parameters affected laminate fiber damage and resin-rich areas, emphasizing the necessity of selecting optimal processing conditions for mechanical qualities. Xiaolong M. et al. [8] used hand lay-up molding to study how processing conditions affected thermoplastic composites’ crystalline and fracture morphology. Processing parameters affected mechanical and crystalline qualities, according to their findings. Dai G. et al. [9] studied how molding temperature, pressure, and holding time affected CF/PEEK cross-laminates’ ILSS. A 20 min holding duration and 400–420 °C temperature range were ideal for molding.

Laser-assisted molding CF/PEEK laminates with different consolidation forces were examined by Qiuyu M. et al. [10]. They found that raising the pressure roller force lowered the void ratio and improved the ILSS, with an average ILSS of 49.95 MPa at 109 N, 9.08% higher than 48 N. Comer A.J. et al. [11] found that AFP laminates had lower ILSS but higher toughness than hot-pressed laminates. This was due to AFP laminates’ high cooling rate and molecular chain movement restrictions. Hu J. et al. [12] examined hot-pressed flat woven carbon fiber fabric-reinforced polyether ether ketone (CFF/PEEK) thermoplastic composites. They studied how molding temperature, pressure, and holding time affect CFF/PEEK composite mechanical properties. A molding temperature of 390 °C reduced resin viscosity and increased fluidity, enabling fiber impregnation and improving composite characteristics.

Kim S.H. and Park C.K. [13] examined how process temperature affects FFR-PP resin impregnation quality. Higher temperatures enhanced resin impregnation but destroyed flax fibers, lowering product quality. Vahid Z. et al. [14] examined how processing temperature (160–240 °C) affected GF-PVC composite strength and microstructure. They found that PVC matrix degradation at 240 °C weakened the fiber-matrix link and reduced flexural characteristics to 39.3 MPa. Jonas B. et al. [15] tested process parameters on consolidation quality. Temperature affected forming quality most, followed by holding time and pressure. Fujihara K. et al. [16] found that lowering the molding temperature and holding time reduced matrix deterioration and improved bending performances in micro-woven CF/PEEK composites. According to Lessard H. et al. [6,17] increasing the mold temperature reduces the cooling rate, improving matrix crystallinity and component mechanical properties.

McCool R. et al. [18] observed that rapid cooling during forming lowers matrix crystallinity, which lowers macroscopic mechanical parameters including bending and interlaminar shear strength. Guangming Dai et al. [19] suggested an all-atom interlayer interface model for unidirectional carbon fiber-reinforced polyether ether ketone (CF/PEEK) composites. MD simulations and experimental validation showed that increasing the forming temperature improves polymer molecular mobility, interlayer diffusion, and bond strength. They also found that interlayer characteristics vary more with temperature than pressure. High-speed filament winding and direct-fired quasi-axial helical filament winding utilizing laser-assisted prepreg thermoplastic tape were shown by Funck R., and Neitzel M. [20]. Heat sources and preheating procedures for winding and molding were compared. In laser-assisted tape winding, stacking layers increase consolidation pressure and contact length, which Amin Hosseini S.M. et al. [21] investigated. Donough M.J. et al. [22] evaluated modeling approaches, material models, predictive analysis, and process parameter optimization for the in situ consolidation of automated fiber placement with thermoplastic prepreg tapes. Present limits and future directions for modeling in situ consolidation processes were reviewed. Flexural properties were used to optimize molding temperature and holding time for continuous CF/PEEK composite laminates by Fujihara K. et al. [16]. High molding temperatures and long holding durations degrade PEEK and diminish mechanical characteristics. Grouve W.J. et al. [23] examined how process factors, material characteristics, and interlaminar bond strength affect laser-assisted tape deployment. The available experimental evidence suggests that the optimal bond quality can be achieved at high speeds and low input power when the laser beam irradiates the tape with a significant portion of the spot area, resulting in a temperature increase in the prepreg that exceeds the substrate temperature, which is below the melt temperature of the PPS.

In the aforementioned study, the automated fiber placement (AFP) technique, despite its flexibility and capacity for producing intricate structures, encountered difficulties in maintaining a continuous fiber lay on the mandrel. This resulted in the deterioration of the mechanical properties of the carbon fiber-reinforced plastic (CFRP). Furthermore, it is challenging to enhance the layup velocity when working with composites that possess cylindrical or axisymmetric geometries.

**Figure 1:**
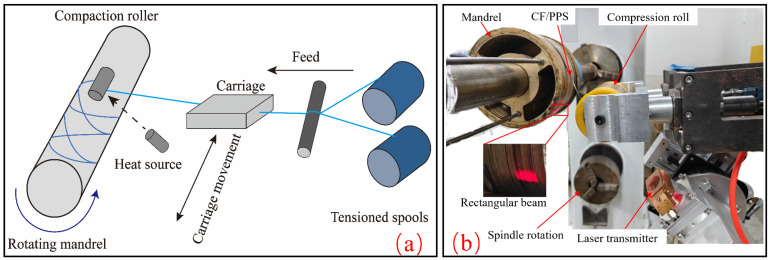
Laser-assisted fiber winding and molding process: (**a**) molding process schematic; (**b**) actual molding process diagram.

In order to mitigate these shortcomings, this paper employs the laser-assisted fiber winding and molding process of CF/PPS as the subject of investigation (shown in Figure 1). A numerical model of laser-heated CF/PPS is established based on the analysis of the thermodynamic properties of CF/PPS. NOL ring tensile tests at different process levels are carried out to explore the influence laws of process parameters on the heating temperature of CF/PPS and the tensile strength of CFRP. The process parameters were optimized using a non-dominated sorting genetic algorithm (NSGA-II) to identify the optimal combination of maximum winding rate and highest tensile strength.

## 2. Materials and Methods

### 2.1. CF/PPS Materials and Thermal Properties Measurement

In this study, T700/PPS unidirectional thermoplastic prepreg (resin mass fraction of 34% ± 3%) produced by Jiangsu Hengbo Composite Materials Co., Ltd. (Danyang, Jiangsu, China) was selected as the material of interest. The thermodynamic properties were measured using a differential scanning calorimeter (DSC214, NETZSCH, Selb, Germany) and a thermogravimetric analyzer (TG209F1, NETZSCH, Germany). In accordance with the stipulations set forth in the GB/T 19466.5-2004 standard [24], differential scanning calorimetry (DSC) was employed to ascertain the melting point temperature of the samples across a temperature range of 25 °C to 400 °C, with varying heating rates of 5 °C/min, 10 °C/min, 15 °C/min, and 20 °C/min. According to the GB/T 19466.4-2004 standard [25], TGA testing was conducted to determine the decomposition temperature of the samples within the range of room temperature to 600 °C at the same heating rates.

### 2.2. Designing Process Solutions

In the laser-assisted CF/PPS prepreg tape winding and molding process, three principal process variables are of consequence: laser power, winding speed, and core mold temperature. Based on the results of the measurements of the melting and decomposition points of CF/PPS, and given that it is challenging for PPS to melt and bond at lower temperatures, while higher temperatures will lead to the degradation of the PPS matrix, this study employs the response surface Box–Behnken design (BBD) to create the three-parameter, three-level winding and molding process of CF/PPS prepregs. The specific levels of the aforementioned process parameters are presented in Table 1.

During laser heating, the laser beam transfers thermal energy to the surface of the thermoplastic composite (CF/PPS), which then spreads via thermal conduction (Equation (1)). The energy distribution is influenced by the laser spot shape, power, movement speed, and material surface properties. The laser power density Q varies over time and space, creating a dynamic heat source that rapidly increases the temperature in the laser focal area, forming a high-temperature region. To optimize computing performance and minimize processing, this work uses a two-dimensional model for simulation. Equation (2) defines the power density Q of the heat source [26].

(1) $$\rho C_{p}\frac{\partial T}{\partial t}=\frac{\partial }{\partial x}\left(k_{x}\frac{\partial T}{\partial x}\right)+\frac{\partial }{\partial y}\left(k_{y}\frac{\partial T}{\partial y}\right)+Q$$

(2) $$Q\left(x,y,t\right)=\frac{P}{a\cdot b}\cdot \chi \left(x,y,t\right)$$

where ρ, $\;C_{p}$, $\;k_{x}$, $k_{y}$ denote the density, specific heat capacity, and anisotropic heat transfer coefficient of the composite material varying with temperature or degree of cure, respectively; *Q* is the laser heating source term. a and b are the dimensions of the rectangular laser spot, p is the laser power, and *χ*(*x*, *y*, *t*) is a function describing the spot’s position and shape over time and space.

For the accurate representation of the heat transfer process, the boundary conditions for convective heat transfer between the surface of the composite material and the surrounding air must be defined (Equation (3)). The convection coefficient is then calculated using Equation (4).

(3) $$-k\nabla T\cdot n=h\left(T-T_{\infty }\right)$$

(4) $$\left\{\begin{array}{c} Re=\frac{\rho uL}{v},\;\mathrm{Pr}=\frac{c_{p}\mu }{k}\\ Nu=0.23Re^{0.8}Pr^{0.4},h=\frac{Nu\cdot k}{L} \end{array}\right.$$

where $T_{\infty }$ is the ambient temperature and h is the convective heat transfer coefficient. The Reynolds number (*Re*), Nusselt number (*Nu*), and Prandtl number (Pr) are defined as follows: *Re* (m/s), *Nu* (m^2^/s), and Pr (J/(kg·K)). The characteristic length (*L*) is in meters, viscosity (μ) in Pa/s, and thermal conductivity (*k*) in W/(m^2^J). CF/PPS related material properties are shown in Table 2.

### 2.3. Testing of Specimens and Determination of Tensile Properties

Following the guidelines of GB/T 1458-2023 [27], we created ring-shaped samples by wrapping CF/PPS prepreg using a DAC500 fiber laser heating system. The samples had a diameter of 150 ± 0.2 mm and a breadth of 6 ± 0.2 mm. The tensile tests were conducted at an ambient temperature using a universal tensile testing machine (model ETM105D, Shenzhen Wance Experimental Equipment Co., Ltd., Shenzhen, China) with a loading rate of 3.0 mm/min. Every batch of samples consisted of six specimens to conduct tensile tests. The unidirectional tensile strength was determined by applying the formula specified in Equation (5).

(5) $$\sigma_{t}=\frac{P_{b}}{2b\cdot h}$$

where $\sigma_{t}$ reinforced composite tensile strength (unit: MPa); $P_{b}$ breaking load (unit: N); *b* specimen width (unit: mm); and *h* specimen thickness (unit: mm).

### 2.4. Methods for Optimization

This study employs the non-dominated sorting genetic algorithm (NSGA-II) to enhance the laser-assisted prepreg tape winding process, addressing the issues of nonlinearity and discontinuity in the design space. The algorithm maximizes tensile strength and rotation rate, thereby demonstrating superior global solution capability and efficiency compared to traditional genetic algorithms.

The optimization process comprises several steps. Initially, objectives and variables must be defined. Then, the population is initialized with combinations of laser power, rotational speed, mandrel temperature, and winding stress. Subsequently, a mathematical model is developed to predict tensile and shear strengths. Finally, the optimization is performed to identify the optimal values. Subsequently, the most efficacious combinations are identified and displayed. The objective of this study is to optimize the material’s tensile strength by adjusting the laser power, rotational speed, mandrel temperature, and winding tension in accordance with Equations (6) and (7).

(6) $$Min\left[-f_{Tensile}-WR\right]$$

(7) $$s.t.\left\{\begin{array}{c} 375\;W\leq LP\leq 525\;W\\ 204.2\;\mathrm{mm}/s\leq WR\leq 510.5\\ 30\;℃\leq MT\leq 90\;℃ \end{array}\right.\mathrm{mm}/s$$

where $f_{Tensile}$ is the maximum tensile strength objective function; W*R* is the rotational speed; L*P* is the laser power; and *MT* is the core mold temperature.

## 3. Results and Discussion

### 3.1. CF/PPS Melting Point and Thermal Decomposition Temperature

**Figure 2:**
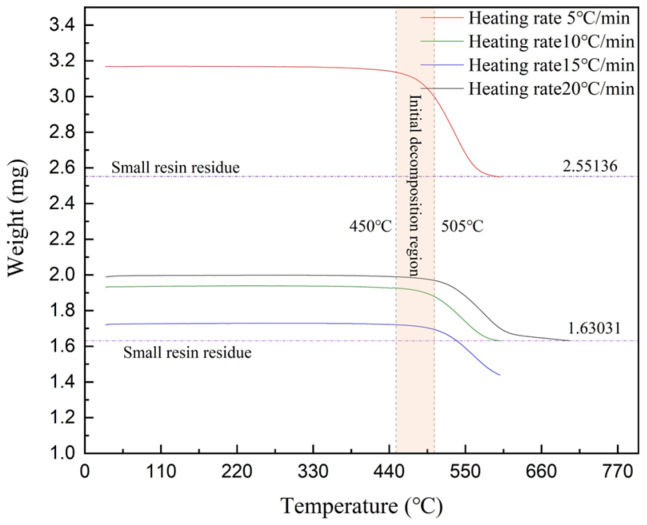
The melting point of CF/PPS under different heating rates.

**Figure 3:**
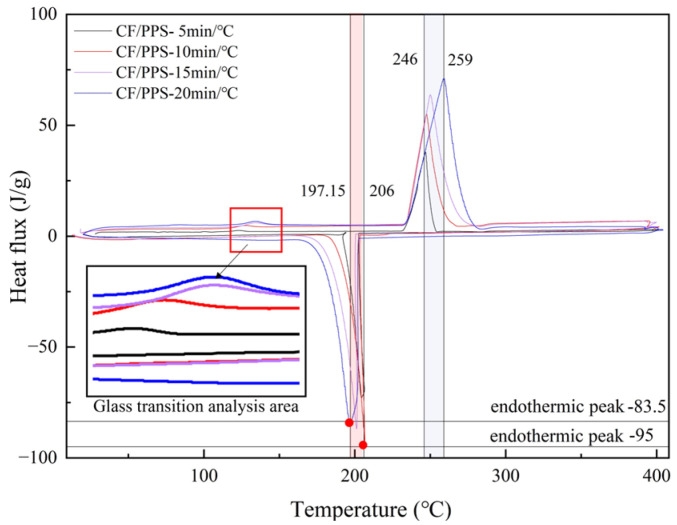
Thermal decomposition temperature of CF/PPS under different heating rates.

Differential scanning calorimetry research demonstrates that the melting point temperature of composites made from carbon fiber-reinforced polyphenylene sulfide increases in proportion to the rate at which they are heated. More precisely, the temperature at which the substance melted increased from 246 °C to 259 °C when the pace at which it was heated was increased from 5 °C/min to 20 °C/min, as shown in Figure 2. The thermal decomposition temperatures of CF/PPS composites also exhibit variations depending on the heating rate, as established through TGA testing. Decomposition initiated within the temperature range of around 450 °C to 505 °C, with heating rates ranging from 5 °C/min to 20 °C/min, as depicted in Figure 3.

The results suggest that the melting point and thermal breakdown temperature of CF/PPS composites are influenced by the pace at which the temperature is increased. This study establishes the CF/PPS melting point at 246 °C and the decomposition temperature at 450 °C to verify the quality of laser-assisted fiber winding molded parts and determine the forming processing window.

### 3.2. Computational Modeling of Laser-Induced Heating in CF/PPS

The present work utilized the COMSOL Multiphysics finite element software Version 6.2 to describe the laser-assisted heating and molding process for CF/PPS winding. The simulation entailed the emission of a laser beam in a homogeneous manner from a rectangular surface, which then proceeded to irradiate both the prepreg tape and the substrate.

It is notable that the power and winding speed of the laser and winding apparatus are expressed as percentages. To ensure consistency with the control of process parameters in actual production, the laser power and winding speed parameters in the BBD scheme are also expressed as percentage values. The winding rates of 20%, 35%, and 50% correspond to linear speeds of 204.2 mm/s, 357.35 mm/s, and 510.5 mm/s, respectively. The convection coefficients for these rates were calculated using Equation (4). The company conducted tests on the laser, which had a power output of 1500 W, and irradiated an area measuring 20 mm × 10 mm. Analysis revealed that 31.75% of the entire beam surface area, equivalent to 6.35 mm × 10 mm, was responsible for effectively heating the prepreg material. Therefore, the laser’s starting intensity was determined to be 5.95 × 10^5^ W/mm^2^, 7.14 × 10^5^ W/mm^2^, and 8.33 × 10^5^ W/mm^2^ at the various rates. The heating duration for the core mold was determined by considering the starting temperature of the solid components, the rate at which the winding was carried out, and the size of the specific area being heated. The heating times corresponding to the winding rates of 20%, 35%, and 50% were 49 ms, 28 ms, and 20 ms, respectively.

**Figure 4:**
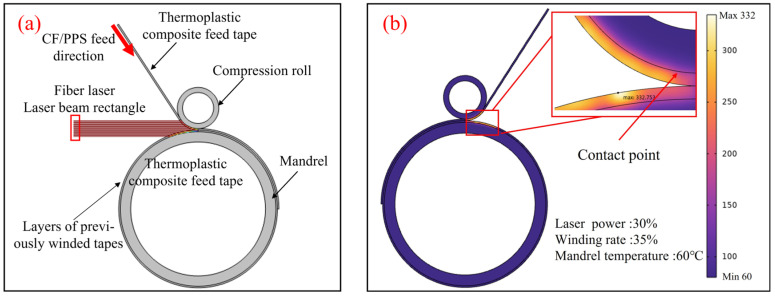
Simulation of laser-assisted CF/PPS wrap-around molding process. (**a**) Numerical modeling; (**b**) temperature analysis.

As illustrated in Figure 4, the CF/PPS prepreg tape is capable of reaching a maximum local heating temperature of 332 °C under specific conditions: a laser power of 30%, a winding rate of 35%, and a core mold temperature of 60 °C. It is crucial to acknowledge that the aforementioned temperature does not account for any potential reduction at the point of contact. A series of simulations were conducted in order to investigate the laser-assisted fiber winding process, with the variables being the laser power, winding rate, and core mold temperature. The Box–Behnken Design response surface method was employed to ascertain the parameter combinations that result in optimal molding effectiveness. The results of the CF/PPS heating temperature calculations are presented in Table 3 for the reader’s convenience.

**Figure 5:**
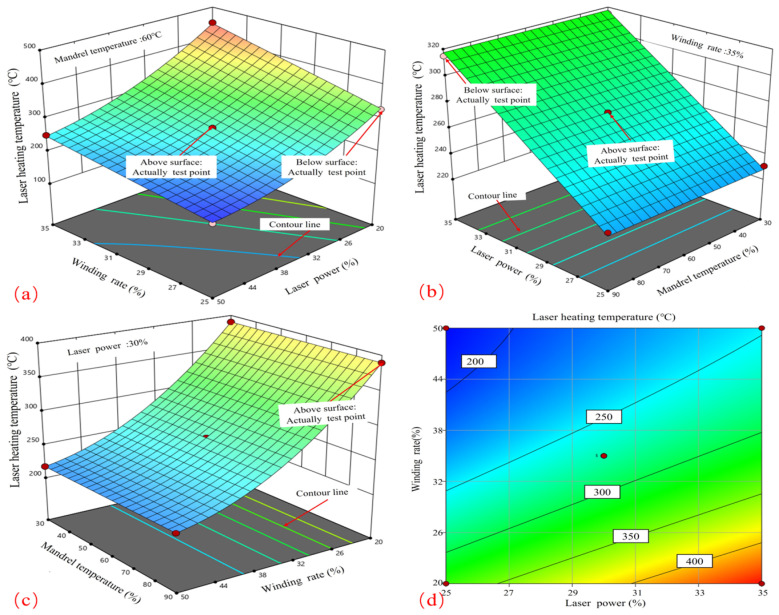
Process coupling effect law on CF/PPS prepreg strip temperature. (**a**) Effect of LP-WR interaction on laser heating temperature; (**b**) Effect of LP-MT interaction on laser heating temperature; (**c**) Effect of WR-MT interaction on laser heating temperature; (**d**) Contours of LP-WR interaction on laser heating temperature.

Figure 5 exhibits the graphical representation of the response surface and contour plots obtained from the computer simulations of laser-induced heating on CF/PPS material. These simulations were conducted using the BBD process scheme. The charts investigate the influence of various parameters, such as laser power, winding rate, and core mold temperature, on the surface temperature of CF/PPS. In Figure 5a, when the winding rate remains constant, the surface temperature shows a nearly linear increase with laser power. The temperature reaches its highest point at 35% laser power. On the other hand, when the laser power remains constant, the effect of the winding rate on the surface temperature demonstrates a progressive rise. Figure 5d demonstrates that the contour plot depicting the relationship between laser power and winding rate is not perfectly circular. The increased ellipticity suggests a substantial interaction between these two parameters.

Figure 5b demonstrates that when the core mold temperature remains constant, the surface temperature increases linearly when the laser power is increased, reaching its highest point at 35% power. Figure 5c demonstrates that the impact of winding rate on surface temperature becomes more significant as the core mold temperature remains constant, but the effect of core temperature increases linearly at a consistent winding rate. To attain the maximum surface temperature, it is important to employ a combination of increased laser power and a swifter winding rate. Considering that the CF/PPS material starts to degrade at a minimum temperature of 450 °C, it is essential to carefully control these parameters to avoid the surface temperature going beyond this limit, particularly when using greater winding rates.

### 3.3. Influence of Process Parameters on Tensile Strength of CF/PPS Composites

#### 3.3.1. Examination of Response Surface Test Results

**Figure 6:**
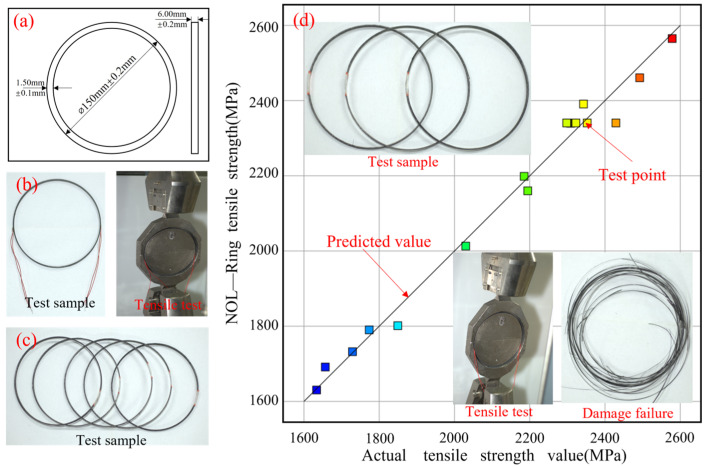
Distribution of predicted and actual values. (**a**) NOL ring standard sample size; (**b**) NOL ring standardized samples and stretching process; (**c**) NOL ring standard sample; (**d**) Typical damage states, tensile values, and predicted results for NOL ring stretching, where the color of the test point changes from blue to red representing progressively larger values.

The results of the NOL ring tests’ tensile strength are displayed in Table 3. In addition, the coefficient of variation (i.e., the ratio of the standard deviation to the mean) was obtained by calculating the data from six valid tests under each group of process combinations. The results show that the maximum coefficient of variation is about 10% and the minimum coefficient of variation is about 4%. Thus, it can be seen that the overall experimental data have a small degree of dispersion and the test data have good reliability. The findings were examined using a comprehensive binomial regression approach with Design Expert 10.0 software, resulting in the creation of a dependable predictive model (refer to Table 4). Figure 6 presents comprehensive information regarding the sample, the methodology employed for testing, and the typical modes of failure. Furthermore, it provides a visual representation of the distribution of projected tensile strength values in comparison to the actual values. The strong correlation and linear association between these values indicate that the prediction model obtained from the response surface analysis is highly reliable. The correspondence between the model predictions and the actual experimental results is exceptionally strong and reliable.

#### 3.3.2. Effect of Single Factors on Tensile Strength

**Figure 7:**
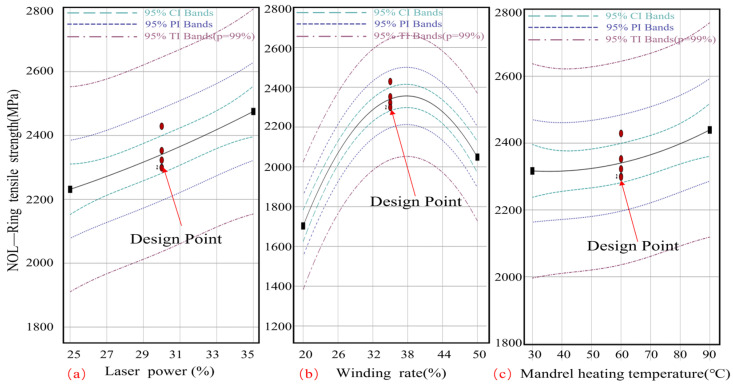
Influence law of single process parameter variation on tensile strength of NOL ring. (**a**) Curve of the effect of LR on the tensile strength of NOL rings; (**b**) Curve of the effect of WR on the tensile strength of NOL rings; (**c**) Curve of the effect of MT on the tensile strength of NOL rings.

As illustrated in Figure 7a, there is a linear correlation between laser power and tensile strength. The data indicate that an increase in laser power results in an enhancement of tensile strength. At a laser power of 25%, the tensile strength is observed to be 2038.31 MPa. A 35% increase in power results in a tensile strength of 500 MPa. As a consequence of the elevated laser intensity, the melting of the resin and the bonding of the fiber to the resin are enhanced, thereby reinforcing the composite.

Figure 7b depicts a parabolic relationship between winding rate and tensile strength. At a winding rate of 20%, the tensile strength is observed to be 250 MPa. At a winding rate of 35%, the tensile strength reaches a maximum of 400 MPa, subsequently declining to 200 MPa at a winding rate of 50%. Higher winding rates initially result in improved fiber alignment and resin distribution, leading to enhanced tensile strength. The application of high winding rates has the potential to increase fiber strain or reduce resin penetration, which in turn may affect the strength of the composite material.

Figure 7c exhibits a concave core mold temperature-tensile strength relationship. The tensile strength is 200 MPa at 30 °C and 350 MPa at 90 °C. Maintaining an optimal core mold temperature is crucial for ensuring homogeneous resin melting and curing, which in turn facilitates enhanced fiber–resin contact. However, elevated temperatures can result in resin degradation, while insufficient temperatures may impede the melting process, thereby impairing the mechanical qualities of the composite. Additionally, the figure illustrates 95% confidence intervals, forecasts, and tolerance intervals as dotted lines. The bands illustrate the probable range of actual and forecast values, confirming the statistical significance of the observed trends and demonstrating the dependability and accuracy of the data.

#### 3.3.3. Process Parameter Interaction

**Figure 8:**
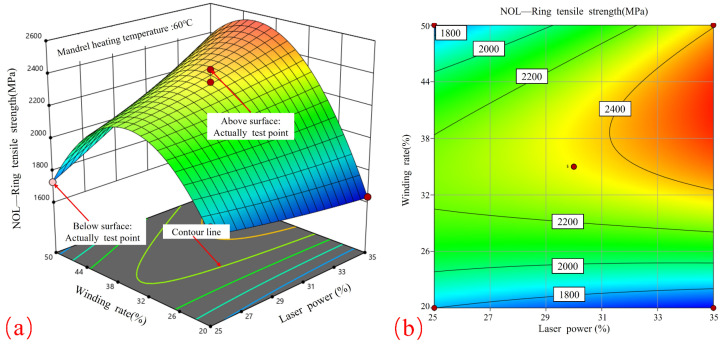
Effect of laser power and winding rate on tensile strength: (**a**) response surface; (**b**) contour plot.

Figure 8 shows the response surface and contour plots that illustrate the relationship between laser power and winding rate in relation to tensile strength. Figure 8a demonstrates that when the laser power is maintained at a constant level, the relationship between the winding rate and tensile strength exhibits a parabolic curve, reaching its maximum value at 35%. Conversely, when the winding rate is maintained at a constant level, the tensile strength will increase in proportion to the laser power, although the rate of growth will be less pronounced. Therefore, in order to achieve the highest possible tensile strength, the optimal combination of process parameters is a winding rate of 35% in conjunction with a higher laser power, both of which fall within the specified process boundaries.

Figure 8b depicts elliptical contours, which indicate a significant correlation between winding tension and curing temperature. The contours display a higher density in proximity to the winding rate axis in comparison to the laser power axis, indicating that the winding rate exerts a more significant influence on tensile strength than the laser power. This finding is in accordance with the observed effects of laser heating on the surface temperature of the core mold. In addition, the results are consistent with the effect of molding rate on the porosity of the product and its quality during CF/PPS placement [28].

**Figure 9:**
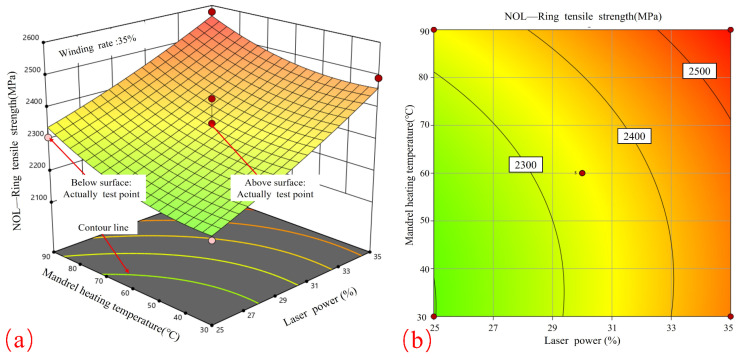
Effect of laser power and core mold temperature on tensile strength: (**a**) response surface; (**b**) contour plot.

Figure 9 illustrates the response surface and contour plots that elucidate the influence of laser power and core mold temperature on tensile strength. Figure 9a illustrates that, when the core mold temperature is maintained at a constant level, the influence of laser power on tensile strength exhibits a nearly linear increase, reaching its maximum at 35%. When the laser power is maintained at a constant level, the impact of the core mold temperature on the tensile strength demonstrates a gradual and consistent increase, reaching its peak at 90 °C. Therefore, the optimal process parameters for attaining the highest tensile strength are an increased laser power and an elevated core mold temperature. The contour plots in Figure 9b exhibit a nearly circular shape, indicating that the relationship between laser power and core mold temperature is relatively insignificant.

**Figure 10:**
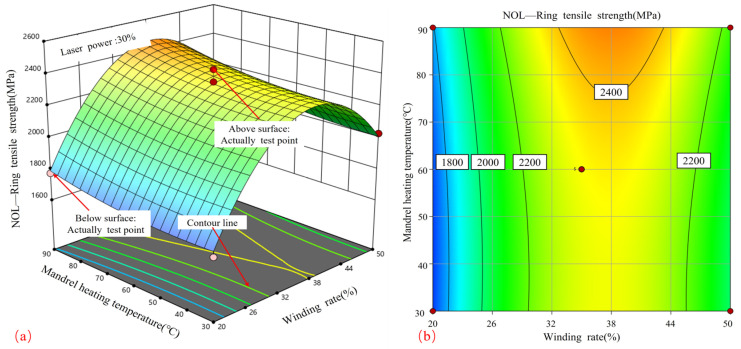
Effect of winding rate and core mold temperature on tensile strength; (**a**) response surface; (**b**) contour plot.

Figure 10 depicts the impact of the winding rate and core mold temperature on tensile strength, as illustrated through the use of response surface and contour plots. Figure 10a illustrates that when the winding rate is maintained at a constant level, the tensile strength demonstrates a predominantly linear increase with the core mold temperature, reaching its maximum value at 90 °C. Conversely, when the core mold temperature is held constant, the effect of the winding rate on tensile strength displays a parabolic pattern, with an initial increase followed by a decrease.

Figure 10b illustrates elliptical contour graphs, which indicate a significant correlation between the winding rate and core mold temperature. The greater density of contour lines surrounding the winding rate axis in comparison to those situated near the core mold temperature axis suggests that the winding rate exerts a more pronounced influence on tensile strength than the core mold temperature. In order to achieve the highest possible tensile strength, it is recommended that a curing temperature of 90 °C and a winding rate ranging from 33% to 45% be employed. Tensile strengths in excess of 2400 MPa were attained using these values.

### 3.4. Composite Process Optimization

#### 3.4.1. Process Parameter Significance Analysis

An analysis of variance and a correlation analysis were conducted to evaluate the impact of laser-assisted winding and molding process parameters on the tensile strength of NOL rings. The results are presented in Table 5, which demonstrates that the effects of different process parameters on tensile strength vary. The results indicate that MT, WR, and LP are significant parameters (*p* < 0.05) for the tensile strength model. The influence is rated as being greatest for WR, followed by LP and then MT. The correlation values for the tensile strength of the NOL ring are 0.28 for LP, 0.4 for WR, and 0.14 for MT.

This ranking is a consequence of the direct correlation between winding speed and the heating time of the CF/PPS at a constant laser power. A reduction in winding speed results in an extended heating period, which increases the temperature of the prepreg tape and may ultimately approach the pyrolysis temperature of the thermoplastic resin PPS. This can lead to a weakening of the bond. Conversely, a higher winding speed reduces the heating time, preventing the surface temperature from reaching the melt temperature and consequently reducing the adhesion of the prepreg layer, which in turn reduces the tensile strength of the NOL ring. Moreover, fluctuations in laser power have a lesser impact than variations in winding rate due to the constrained process window.

The correlation coefficient for the interaction between LP and WR is 0.51, indicating that enhancing this interaction could significantly enhance material strength. In order to achieve the greatest enhancement in material properties, it is essential to devote considerable attention to the winding rate and its synergistic effect with laser power during process optimization.

#### 3.4.2. Molding Process Parameter Validation and Optimization

For the optimization procedure, Isight’s non-dominated sorting genetic algorithm (NSGA-II) was employed. The parameters were as follows: a variance distribution index of 20.0, a crossover chance of 0.9, a population size of 60, and several generations of 100. In ensuring the highest winding rate and tensile strength, the optimization results after 6001 iterations show a laser power of 525 W, a winding rate of 424.6 mm/s, a mandrel temperature of 90.0 °C, and a tensile strength of 2647.8 MPa.

**Figure 11:**
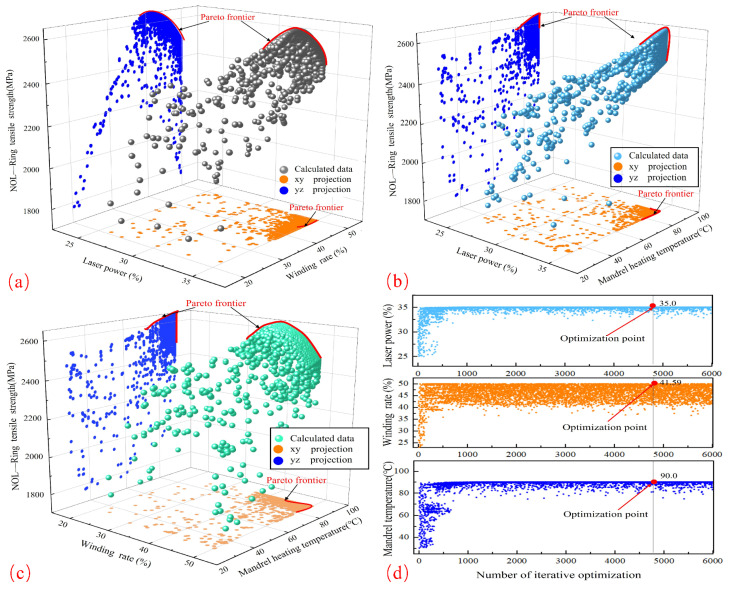
Pareto frontiers for the response variables: (**a**) LP vs. WR vs. NTS; (**b**) LP vs. MT vs. NTS; (**c**) WR vs. MT vs. NTS; (**d**) LP, WR, and MT iterations.

The Pareto fronts for laser power vs winding rate, laser power versus core temperature, and core temperature versus winding rate are shown in Figure 11a, Figure 11b, and Figure 11c, respectively. The impacts of these parameter combinations on tensile strength and the optimization process are clearly shown by these figures and projections on the xy and xz planes. The results show that by carefully modifying these process parameters, the material’s tensile strength may be greatly increased.

The laser power, winding rate, and mandrel temperature were tuned, and experiments were carried out using these optimized parameters to confirm the accuracy of the tensile strength predictions for NOL rings produced by response surface optimization. Table 6 records the test outcomes. An excellent fit of the model is indicated by the minor relative errors in the tensile strength between the actual and projected values (3.2% vs. 2.8%, respectively).

The results of the experiment show that, under optimized conditions, the tensile properties of NOL rings meet the expected levels, indicating the validity of the optimal process parameters determined by the satisfaction function method. Any detected differences could be the result of flaws in the experimental data or constraints in the optimization model. In real-world applications, the optimization model algorithm, the experimental design technique, or a larger sample size can all be improved to increase the dependability of the optimization results.

## 4. Conclusions

To enhance the tensile properties of thermoplastic composites, this study explored the relationship between laser-assisted fiber winding process parameters—laser power, winding rate, and core mold temperature—and the tensile strength of NOL rings using the Box–Behnken design response surface methodology. A numerical model was developed to analyze the influence of process variables on CF/PPS surface temperature during molding. Additionally, a mathematical model was constructed to predict the mechanical properties of NOL rings, identifying significant interactions between different process parameters through response surface and contour plots. Experimental validation confirmed the optimization results, demonstrating that the optimized process parameters significantly improved the tensile strength of NOL rings. Specifically, the optimal parameters were 525 W laser power, 424.7 mm/s winding speed, and 90 °C core mold temperature. The tensile strength of the optimized NOL ring reached 2647.80 MPa, significantly higher than the non-optimized result, underscoring the effectiveness and practicality of this optimization method.

The principal findings can be summarized as follows:

1. The objective of this study is to investigate the influence of process parameters on surface temperature. The surface temperature of the prepreg tape is significantly affected by the laser power, winding rate, and core mold temperature. The order of influence is as follows: the interaction between laser power and winding rate is the most significant factor, followed by winding rate, laser power, and core mold temperature.
2. The effect of process parameters on tensile strength is as follows: The tensile strength of NOL rings is significantly influenced by the laser power, winding rate, and core mold temperature. The response surface and contour plots revealed a correlation coefficient of 0.51 for the interaction between laser power and winding rate, indicating that optimizing their combination is crucial for enhancing tensile strength.
3. The optimization results are as follows: The optimized parameters, which consisted of 35% laser power (525 W), 41% winding rate (418.6 mm/s), and 90 °C core mold temperature, resulted in the highest tensile strength for NOL rings, exhibiting a significant improvement over the unoptimized process parameters.

## Figures and Tables

**Table 1:** Parameter levels of thermoplastic prepreg winding process.

Parameter Level	Laser Power/(W)	Winding Speed/(mm/s)	Mold Temperature/(°C)
High level	525	510.5	90
Low level	375	204.2	30

Note: The maximum laser power is 1500 W; the maximum winding rate is 130 r/min.

**Table 2:** CF/PPS key material properties.

ρ	$k_{x}$	$k_{y}$	$C_{p}$	Wavelength	Actual Laser Spot Size
1570 kg/m^3^	6.0 W/(m·K)	0.7 W/(m·K)	1260 J/(kg·K)	960 nm	6.35 × 10 mm^2^

**Table 3:** CF/PPS heating temperature and NOL ring tensile test results.

Order	Laser Power Percentage/%	Percent Winding Rate/%	Mandrel Heating Temperature/°C	Heating Temperature/°C	Tensile Strength/MPa	Coefficient of Variation%
1	30	50	90	307.40	2194.77	9.70
2	35	20	60	511.39	1633.83	7.94
3	35	50	60	310.30	2342.89	3.58
4	25	35	90	320.63	2308.31	7.66
5	30	35	60	332.75	2352.64	6.98
6	25	35	30	260.63	2185.23	3.50
7	30	35	60	332.75	2322.68	6.17
8	30	20	30	419.77	1656.85	8.16
9	35	35	90	404.88	2558.46	6.61
10	25	50	60	244.50	1729.04	4.65
11	30	20	90	479.77	1773.57	9.61
12	30	35	60	332.75	2298.38	7.69
13	25	20	60	388.14	1849.45	9.56
14	30	35	60	332.75	2299.93	7.61
15	35	35	30	344.88	2492.17	8.09
16	30	35	60	332.75	2428.98	7.08
17	30	50	30	247.40	2029.85	8.28

**Table 4:** Response surface regression model.

Forecasting Function		Mean Square	F-Value	*p*-Value
Stretch fitting equation	f1 = 2017.18 − 99.38x_1_ + 71.48x_2_2.08x_3_ + 2.76x_1_x_2_ − 0.061x_1_x_3_ − 0.027x_2_x_3_ + 0.51x_1_^2^ − 2.06x_2_^2^ + 0.04x_3_^2^	1.634 × 10^5^	52.84	<0.0001

**Table 5:** Analysis of variance results of NOL ring tensile strength fitting model.

Model	Tensile Strength/MPa		Correlation Coefficient
	F-Value	*p*-Value	
LP	38.45	0.0004	0.28
WR	77.30	<0.0001	0.40
MT	9.75	0.0168	0.14
LP*WR	55.62	0.0001	0.51
LP*MT	0.11	0.7505	0.22
WR*MT	0.19	0.6778	0.36
LP^2^	0.22	0.6515	---
WR^2^	293.78	<0.0001	---
MT^2^	1.94	0.2064	---

**Table 6:** Optimization results and comparison of non-optimal solutions.

	Laser Power Percentage/%	Percent Winding Rate/%	Mandrel Heating Temperature/℃	Tensile Strength/MPa	Relative Error/%
Optimization solution	35.0	41.6	90.0	2647.80	——
Measured value#1	35.0	41.0	90.0	2564.23	3.2%
Measured value#2	35.0	41.0	90.0	2571.51	2.8%

## Data Availability

Dataset available on request from the authors.

## References

[1] Van Hoa S., Duc Hoang M., Simpson J. (2017). Manufacturing procedure to make flat thermoplastic composite laminates by automated fiber placement and their mechanical properties. J. Thermoplast. Compos. Mater..

[2] Wanigasekara C., Oromiehie E., Swain A., Prusty B.G., Nguang S.K. (2020). Machine learning based predictive model for AFP-Based unidirectional composite laminates. IEEE Trans. Ind. Inform..

[3] Oromiehie E., Garbe U., Gangadhara Prusty B. (2020). Porosity analysis of carbon fiber reinforced polymer laminates manufactured using automated fiber placement. Compos. Mater..

[4] Qureshi Z., Swait T., Scaife R., El-Dessouky H.M. (2014). In situ consolidation of thermoplastic prepreg tape using automated tape placement technology: Potential and possibilities. Compos. Part B Eng..

[5] Zheng B., Li M., Deng T., Zhou H., Huang Z., Zhou H., Li D. (2019). Process-structure-property relationships of thermoformed woven carbon-fiber-reinforced polyether ether ketone composites. Polym. Compos..

[6] Lessard H., Lebrun G., Benk A., Pham X.T. (2015). Influence of process parameters on the thermostamping of a [0/90]_12_ carbon/polyether ether ketone laminate. Compos. Part A Appl. Sci. Manuf..

[7] Oromiehie E., Gain A.K., Prusty B.G. (2021). Processing parameter optimisation for automated fibre placement (AFP) manufactured thermoplastic composites. Compos. Struct..

[8] Ma X.L., Wen L.H., Wang S.Y., Xiao J.Y., Li W.H., Hou X. (2023). Inherent relationship between process parameters, crystallization and mechanical properties of continuous carbon fiber reinforced PEEK composites. Def. Technol..

[9] Dai G., Zhan L., Guan C., Huang M. (2020). The effect of moulding process parameters on interlaminar properties of CF/PEEK composite laminates. High Perform. Polym..

[10] Miao Q., Dai Z., Ma G., Niu F., Wu D. (2021). Effect of consolidation force on interlaminar shear strength of CF/PEEK laminates manufactured by laser-assisted forming. Compos. Struct..

[11] Comer A.J., Ray D., Obande W.O., Jones D., Lyons J., Rosca I., O’higgins R.M., McCarthy M.A. (2015). Mechanical characterisation of carbon fibre–PEEK manufactured by laser-assisted automated-tape-placement and autoclave. Compos. Part A Appl. Sci. Manuf..

[12] Hu J., Zhang H., Li S., Ji C., Chen S., Zhou Z., Wang B. (2022). Process parameter–mechanical property relationships and influence mechanism of advanced CFF/PEEK thermoplastic composites. Polym. Compos..

[13] Kim S.H., Park C.K. (2017). Direct impregnation of thermoplastic melt into flax textile reinforcement for semi-structural composite parts. Ind. Crops Prod..

[14] Vahid Z., Moslemi Naeini H., Bahramian A.R., Abdollahi H., Behravesh A.H. (2016). Investigation of the effect of processing temperature on the elastic and viscoelastic properties of PVC/fiberglass composite laminates. Modares Mech. Eng..

[15] Jonas B., Roshan S. (2000). Effect of processing parameters on consolidation quality of GF/PP commingled yarn based composites. J. Thermoplast. Compos. Mater..

[16] Fujihara K., Huang Z., Ramakrishna S., Hamada H. (2004). Influence of processing conditions on bending property of continuous carbon fiber reinforced PEEK composites. Compos. Sci. Technol..

[17] McCool R., Murphy A., Wilson R., Jiang Z., Price M., Butterfield J., Hornsby P. (2012). Thermoforming carbon fibre-reinforced thermoplastic composites. Proc. Inst. Mech. Eng. Part L J. Mater. Des. Appl..

[18] Gao S.L., Kim J.K. (2015). Cooling rate influences in carbon fibre/PEEK composites. Part 1. Crystallinity and interface adhesion. Compos. Part A Appl. Sci. Manuf..

[19] Dai G., Zhan L., Ma B., Guan C., Huang M. (2023). Effect of process parameters on interlaminar properties of thermoplastic composite: Molecular dynamics simulation and experimental verification. Polymer.

[20] Funck R., Neitzel M.P. (1995). Improved thermoplastic tape winding using laser or direct-flame heating. Compos. Part A Appl. Sci. Manuf..

[21] Hosseini S.A., Baran I., van Drongelen M., Akkerman R. (2021). On the temperature evolution during continuous laser-assisted tape winding of multiple C/PEEK layers: The effect of roller deformation. Int. J. Mater. Form..

[22] Donough M.J., Shafaq S., John N.A., Philips A.W., Prusty B.G. (2022). Process modelling of In-situ consolidated thermoplastic composite by automated fibre placement a review. Compos. Part A Appl. Sci. Manuf..

[23] Grouve W.J., Warnet L.L., Rietman B., Visser H.A., Akkerman R. (2013). Optimization of the tape placement process parameters for carbon–PPS composites. Compos. Part A Appl. Sci. Manuf..

[24] (2004). Plastics Thermal Analysis Part 5: Differential Scanning Calorimetry (DSC).

[25] (2004). Plastics Thermal Analysis Part 4: Thermogravimetric Analysis (TGA).

[26] Esselink F.S., Hosseini S.M., Baran I., Akkerman R. (2020). Optimization of laser-assisted tape winding/placement process using inverse optical model. Scope Procedia Manuf..

[27] (2023). Test Method for Mechanical Properties of Ring of Filament-Winding Reinforced Composites.

[28] Zhao D., Chen J., Zhang H., Liu W., Yue G., Pan L. (2022). Effects of processing parameters on the performance of carbon fiber reinforced polyphenylene sulfide laminates manufactured by laser-assisted automated fiber placement. J. Compos. Mater..

